# Association between handgrip strength and hypertension in children and adolescents: an analysis of the National Health and Nutrition Examination Survey 2011–2014

**DOI:** 10.3389/fped.2025.1559556

**Published:** 2025-04-14

**Authors:** Huanhuan Li, Hongmei Gu

**Affiliations:** ^1^ Department of Electrocardiogram, Huanggang Central Hospital, Huanggang, Hubei, China; ^2^ School of Public Health, Mudanjiang Medical University, Mudanjing, Heilongjiang, China

**Keywords:** handgrip strength, hypertension, HGS asymmetry ratio, children and adolescents, NHANES

## Abstract

**Aim:**

Handgrip strength (HGS), a measure of muscle strength, has been reported to be associated with the risk of hypertension in adults. This study intended to assess the relationship of relative HGS (rHGS) and HGS asymmetry ratio with elevated blood pressure and hypertension in children and adolescents.

**Methods:**

This cross-sectional study included children and adolescents aged 6–19 years with HGS and blood pressure measurements in the 2011–2014 National Health and Nutrition Examination Survey (NHANES) dataset. The relationships of rHGS and HGS asymmetry ratio with elevated blood pressure and hypertension were assessed using weighted logistic regression models and described as odds ratio (OR) with 95% confidence interval (CI). Subgroup analysis was conducted according to age (<13, ≥13 years) and gender (male, female).

**Results:**

In total, 3,736 children and adolescents were included in the study, of whom 509 (13.75%) had elevated blood pressure and 188 (4.72%) had hypertension. High rHGS levels were related to lower odds of elevated blood pressure (OR = 0.52, 95%CI, 0.33–0.81) and hypertension (OR = 0.34, 95%CI, 0.18–0.66). In addition, children and adolescents with HGS asymmetry ratio of >30.0% had higher odds of elevated blood pressure (OR = 2.14, 95%CI, 1.27–3.61) and hypertension (OR = 3.02, 95%CI, 1.42–6.42). Subgroup analyses demonstrated that the relationship between high rHGS levels and lower odds of elevated blood pressure and hypertension did not differ by age or sex, whereas the association between HGS asymmetry ratio of >30.0% and higher odds of elevated blood pressure and hypertension was found only in children ≥13 years and males.

**Conclusion:**

High rHGS levels were associated with lower odds of elevated blood pressure and hypertension in children and adolescents, whereas an HGS asymmetry ratio of >30.0% was related to higher odds of elevated blood pressure and hypertension.

## Introduction

Hypertension is a major risk factor for the development of cardiovascular disease in people of all ages (1, 2). Exposure to hypertension in childhood and adolescence has been found to be significantly associated with an increased risk of cardiovascular, cerebrovascular, and other chronic diseases in adulthood (3). Epidemiologic studies showed that approximately 14% of U.S. adolescents aged 12–19 had elevated blood pressure or hypertension during 2013–2016 (4). In addition, hypertension is difficult to diagnose in children and adolescents, and its true prevalence may be underestimated (5). Prevention and control of hypertension in children and adolescents is key to primary prevention of cardiovascular disease.

Skeletal muscle, the organ with the largest mass in the body, significantly influences metabolic processes, and skeletal muscle health is an important cardiometabolic marker (6). Handgrip strength (HGS) is a simple and reliable physiologic indicator of skeletal muscle strength in clinical settings (7). Several studies have reported that high HGS may be associated with a reduced risk of hypertension in adults (8, 9). Other studies have found that high HGS may be positively associated or not associated with the risk of hypertension in adults (10, 11). These inconsistent results may be influenced by body mass index (BMI), which has a significant effect on HGS values (10, 12). Relative HGS (rHGS) and HGS asymmetry ratio are both indicators of the level of HGS, with rHGS adjusting for BMI on the basis of absolute HGS and HGS asymmetry ratio responding to the difference in HGS between two hands (13, 14). rHGS and HGS asymmetry ratio have been found to be significantly related to the risk of metabolism-related diseases such as hypertension in adults as well as in the elderly population (8, 15). However, the relationship between HGS-related indicators and the risk of hypertension remains unclear in children and adolescent populations. Therefore, this study intended to investigate the association of rHGS and HGS asymmetry ratio with elevated blood pressure and hypertension in children and adolescents.

## Methods

### Study design and data sources

The data analyzed for this cross-sectional study were obtained from the National Health and Nutrition Examination Survey (NHANES) dataset from 2011 to 2014. The NHANES survey is conducted by the National Center for Health Statistics (NCHS) to evaluate the health and nutrition status of the United States non-hospitalized population (https://wwwn.cdc.gov/nchs/nhanes/AnalyticGuidelines.aspx). The NHANES survey collects data from participants through interviews and physical examinations in survey cycles every 2 years. Data in NHANES include demographic, dietary, socioeconomic, health-related, medical, physiologic measurements, and laboratory tests. Since HGS measurement data were only available in two survey cycles, 2011–2012 and 2013–2014, only NHANES data from 2011 to 2014 were analyzed in this study. Participants aged 6–19 years with HGS and blood pressure measurements were included. The exclusion criteria were as follows: (1) participants had any pain, aching or stiffness in right/left hand in the past 7 days; (2) participants with missing information on key covariates (e.g., BMI). The NHANES protocols were approved by the NCHS Research Ethics Review Board and written informed consent was obtained from each participant.

### Outcomes

The outcomes were elevated blood pressure and hypertension. The diagnosis of elevated blood pressure and hypertension was based on the criteria of the American Academy of Pediatrics (16): (1) for children aged <13 years, elevated blood pressure was defined as systolic blood pressure (SBP) or diastolic blood pressure (DBP) ≥90th percentile blood pressure; hypertension was defined as SBP or DBP ≥95th percentile blood pressure; (2) for children aged ≥13 years, elevated blood pressure was defined as SBP/DBP ≥120/80 mmHg; hypertension was defined as SBP/DBP ≥130/80 mmHg. The 90th and 95th percentiles were derived by using quantile regression on the basis of normal-weight children (BMI <85th percentile).

### Measurement of HGS

Muscle strength was measured through a HGS test using a handgrip dynamometer (17). After the examiner explained and demonstrated the test protocol to the participant, adjusted the grip size of the dynamometer, and the participant practiced the test, the participant was asked to squeeze the dynamometer as hard as possible with one hand while exhaling to avoid a buildup of pressure in the chest. This test was then repeated on the other hand. Each hand was tested three times alternately. The combined HGS was calculated as the sum of the maximum readings from each hand. The rHGS was calculated as: the combined HGS/BMI. The HGS asymmetry ratio was calculated as: [(maximumHGSofthedominanthand/maximumHGSof thenon-dominanthand)−1]×100%. The rHGS values were divided into three groups based on tertiles according to male and female, respectively [male: <2.13 (low), 2.13–3.22 (median), ≥3.22 (high); female: <1.86 (low), 1.86–2.4 (median), ≥2.4 (high)]. The HGS asymmetry ratio was categorized into four groups according to previous studies (18): 0%–10%, 10.1%−20.0%, 20.1%−30.0%, and >30.0%.

### Data collection

Participant data were collected including age, gender (male, female), race (White, Black, others), education level (below high school, high school, above high school), family poverty-to-income ratio (PIR) (<1.3, ≥1.3, unknown), screen time (<5, ≥5 h), physical activity (non-ideal, ideal), birth weight (<5.5 pounds, ≥5.5 lbs, unknown), household smokers (no, yes, unknown), family education (below high school, high school, above high school, unknown), total cholesterol, direct high density lipoprotein cholesterol (HDL-C), cotinine (<0.05, ≥0.05 μg/L, unknown), Healthy Eating Index (HEI)-2020, HGS, rHGS, and HGS asymmetry ratio.

### Statistical analysis

Continuous data were described as mean and standard error (S.E), and weighted *t*-test was used for comparisons between groups. Categorical data were described as the numbers and composition ratio [*N* (%)], and the Chi-square test was used for comparisons between multiple groups. Weighted variables (SDMVPSU, SDMVSTRA, WTINT2YR) from the NHANES database were used for statistical analysis.

A few variables (education level, physical activity, direct HDL-C, total cholesterol, and HEI-2020) had missing values, and multiple interpolation method was used to interpolate the missing values. Difference analysis showed no statistically significant differences before and after the interpolation of missing variables (Supplementary Table 1). Weighted univariable logistic regression model was applied to screen for confounders related to elevated blood pressure and hypertension, respectively. Variables with *P* < 0.05 in the univariable model were adjusted as confounders in the multivariable model (Supplementary Tables 2, 3). The associations of rHGS and HGS asymmetry ratio with elevated blood pressure and hypertension were assessed using weighted univariable and multivariable logistic regression models and expressed as odds ratio (OR) with 95% confidence interval (CI). Moreover, the non-linear relationship of rHGS with elevated blood pressure and hypertension was explored using restricted cubic spline (RCS). Subgroup analysis was conducted according to age (<13, ≥13 years) and gender (male, female). Statistical analyses were completed using SAS 9.4 software (SAS Institute, Cary, NC). Statistical significance was defined as *P* < 0.05.

## Results

### Characteristics of populations

**Figure 1:**
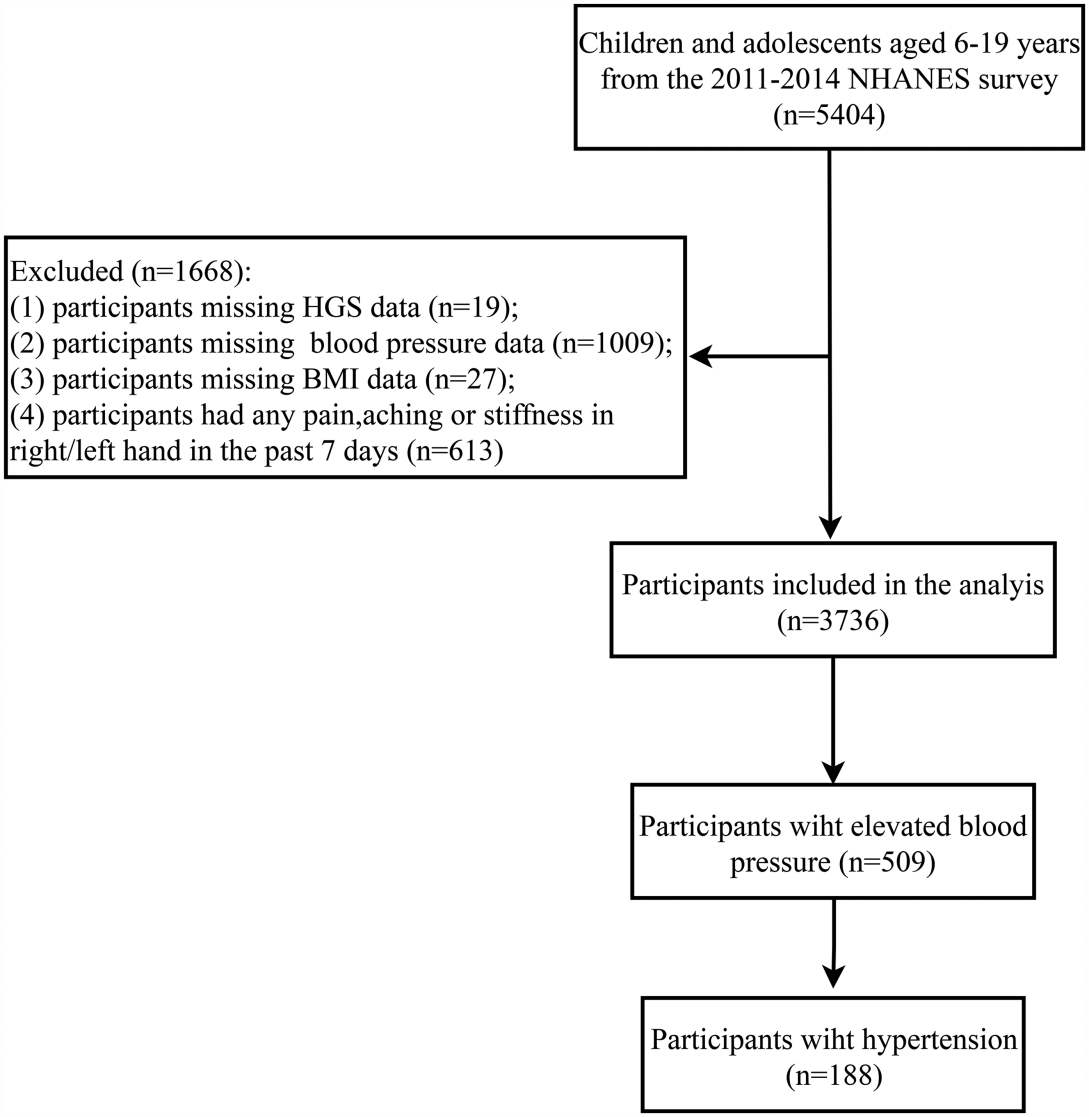
Screening flowchart for the population. NHANES, the National Health and Nutrition Examination Survey; HGS, handgrip strength; BMI, body mass index.

A total of 5,404 children and adolescents aged 6–19 years were recorded in the 2011–2014 NHANES survey. After excluding 1,668 participants, 3,736 participants were included in the analysis (Figure 1). The characteristics of participants according to elevated blood pressure were shown in Table 1. Moreover, the characteristics of participants according to hypertension were presented in Supplementary Table 4. There were 509 (13.75%) participants with elevated blood pressure and 188 (4.72%) participants with hypertension. The mean age was 13.44 (0.10) years and 1,876 (50.28%) participants were male. The mean HGS and rHGS were 28.38 (0.37) kg and 2.44 (0.03), respectively. There were 2,206 (59.62%) participants with HGS asymmetry ratio of 0%–10%, 1,112 (29.16%) with HGS asymmetry ratio of 10.1%–20.0%, 320 (8.86%) with HGS asymmetry ratio of 20.1%–30.0%, and 98 (2.36%) with HGS asymmetry ratio of >30.0%.

**Table 1:** Characteristics of children and adolescents according to elevated blood pressure.

Variables	Total (*n* = 3,736)	Non-elevated blood pressure (*n* = 3,227)	Elevated blood pressure (*n* = 509)	*P*
Age (years), mean (S.E)	13.44 (0.10)	13.32 (0.10)	14.21 (0.22)	<0.001
Gender, *n* (%)				<0.001
Male	1,876 (50.28)	1,535 (47.47)	341 (67.90)	
Female	1,860 (49.72)	1,692 (52.53)	168 (32.10)	
Race, *n* (%)				0.033
White	932 (54.92)	809 (55.52)	123 (51.18)	
Black	1,015 (14.22)	844 (13.52)	171 (18.61)	
Others	1,789 (30.86)	1,574 (30.97)	215 (30.22)	
Education level, *n* (%)				0.028
Below high school	3,378 (89.05)	2,945 (89.97)	433 (83.30)	
High school	199 (5.87)	157 (5.44)	42 (8.59)	
Above high school	159 (5.08)	125 (4.60)	34 (8.11)	
PIR, *n* (%)				0.225
<1.3	1,606 (31.94)	1,365 (31.27)	241 (36.15)	
≥1.3	1,861 (61.93)	1,623 (62.49)	238 (58.44)	
Unknown	269 (6.12)	239 (6.23)	30 (5.41)	
Screen time (hours)				0.252
<5	2,573 (70.97)	2,247 (71.55)	326 (67.35)	
≥5	1,163 (29.03)	980 (28.45)	183 (32.65)	
Physical activity, *n* (%)				0.369
Non-ideal	1,397 (35.27)	1,218 (35.58)	179 (33.29)	
Ideal	2,339 (64.73)	2,009 (64.42)	330 (66.71)	
Birth weight (pounds), *n* (%)				<0.001
<5.5	310 (6.28)	268 (6.19)	42 (6.85)	
≥5.5	2,247 (59.46)	2,008 (62.01)	239 (43.44)	
Unknown	1,179 (34.27)	951 (31.80)	228 (49.71)	
Household smokers, *n* (%)				0.133
No	3,058 (83.00)	2,652 (83.61)	406 (79.17)	
Yes	459 (11.09)	387 (10.59)	72 (14.23)	
Unknown	219 (5.91)	188 (5.80)	31 (6.59)	
Family education, *n* (%)				0.432
Below high school	925 (19.28)	797 (19.14)	128 (20.17)	
High school	828 (20.85)	700 (20.40)	128 (23.61)	
Above high school	1,866 (56.85)	1,630 (57.58)	236 (52.27)	
Unknown	117 (3.02)	100 (2.87)	17 (3.95)	
Total cholesterol (mg/dl), Mean (S.E)	157.39 (0.69)	156.74 (0.75)	161.47 (1.92)	0.030
Direct HDL-C (mg/dl), Mean (S.E)	52.06 (0.36)	52.37 (0.39)	50.14 (0.61)	0.002
Cotinine (μg/L), *n* (%)				<0.001
<0.05	1,869 (52.10)	1,649 (53.62)	220 (42.54)	
≥0.05	1,382 (35.87)	1,148 (33.91)	234 (48.21)	
Unknown	485 (12.03)	430 (12.47)	55 (9.25)	
HEI-2020, mean (S.E)	46.18 (0.33)	46.28 (0.35)	45.54 (0.90)	0.448
HGS, mean (S.E)	28.38 (0.37)	27.67 (0.35)	32.85 (0.90)	<0.001
rHGS, mean (S.E)	2.44 (0.03)	2.43 (0.03)	2.56 (0.06)	0.016
rHGS, *n* (%)				0.122
Low	1,409 (33.00)	1,193 (32.22)	216 (37.88)	
Median	1,245 (33.97)	1,107 (34.80)	138 (28.75)	
High	1,082 (33.03)	927 (32.98)	155 (33.37)	
HGS asymmetry ratio, *n* (%)				0.234
0%–10%	2,206 (59.62)	1,914 (59.70)	292 (59.12)	
10.1%–20.0%	1,112 (29.16)	963 (29.44)	149 (27.42)	
20.1%–30.0%	320 (8.86)	271 (8.76)	49 (9.49)	
>30.0%	98 (2.36)	79 (2.11)	19 (3.97)	

PIR, family poverty-to-income ratio; HDL-C, high density lipoprotein cholesterol; HEI-2,020, healthy eating index-2,020; HGS, handgrip strength; rHGS, relative HGS [male (tertiles): <2.13 (low), 2.13–3.22 (median), ≥3.22 (high); female (tertiles): <1.86 (low), 1.86–2.4 (median), ≥2.4 (high)].

### Relationship of rHGS and HGS asymmetry ratio with elevated blood pressure and hypertension in children and adolescents

**Figure 2:**
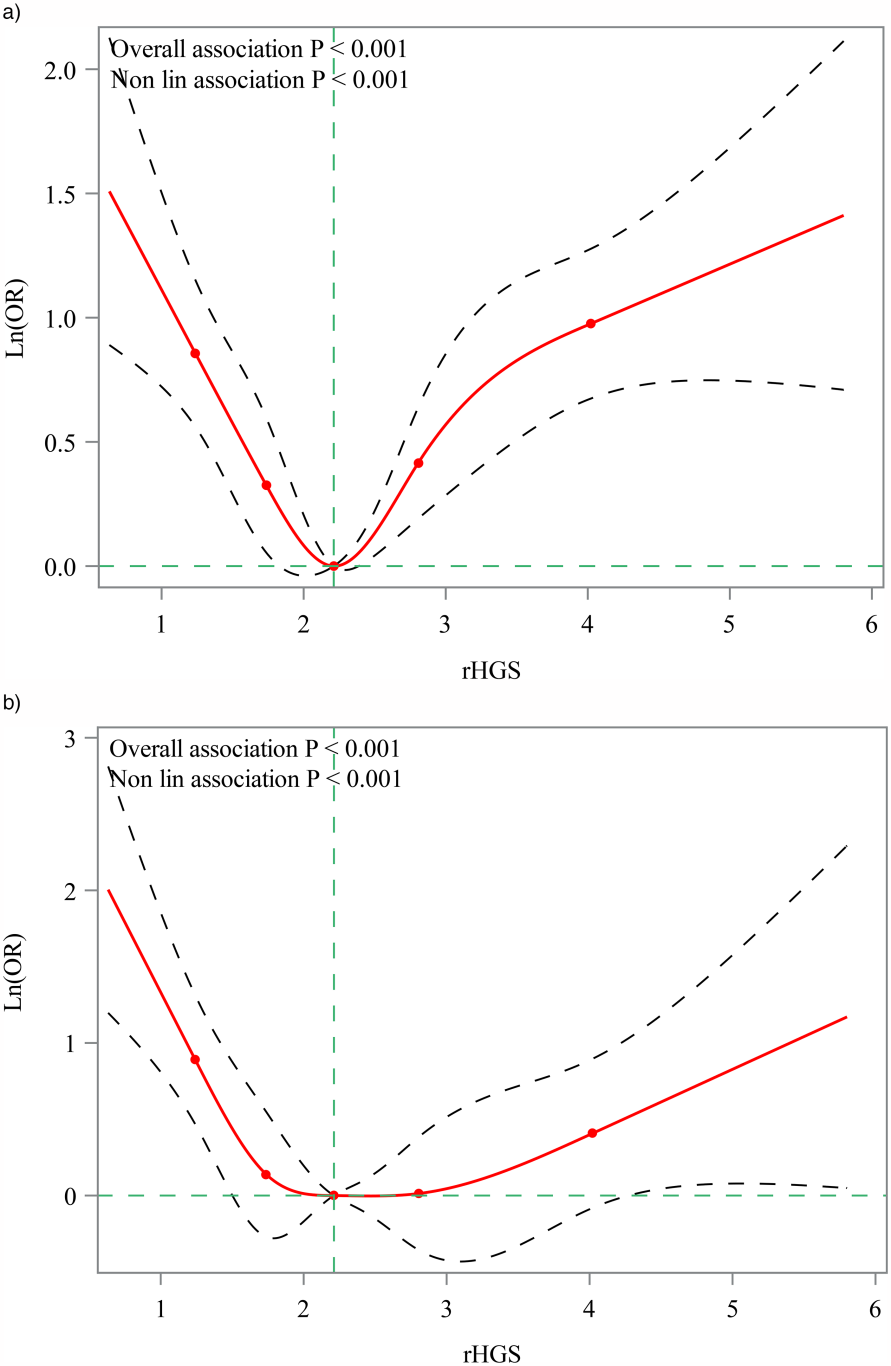
Restricted cubic spline (RCS) for the non-linear relationship of rHGS with elevated blood pressure and hypertension. **(a)** Association between rHGS and elevated blood pressure; **(b)** association between rHGS and hypertension. rHGS, relative handgrip strength; OR, odds ratio.

The associations between HGS and elevated blood pressure and hypertension in children and adolescents were presented in Table 2. Children with median (OR = 0.52, 95%CI, 0.32–0.85) and high (OR = 0.52, 95%CI, 0.33–0.81) rHGS levels were related to lower odds of elevated blood pressure compared with children with low rHGS levels. For hypertension, median (OR = 0.55, 95%CI, 0.34–0.89) and high (OR = 0.34, 95%CI, 0.18–0.66) rHGS levels were also correlated with lower odds of hypertension compared with low rHGS levels. In addition, children with HGS asymmetry ratio of >30.0% had higher odds of elevated blood pressure (OR = 2.14, 95%CI, 1.27–3.61) and hypertension (OR = 3.02, 95%CI, 1.42–6.42) compared with children with HGS asymmetry ratio of 0%–10%, but not for children with HGS asymmetry ratio of 10.1%–20.0% and 20.1%–30.0% (*P* > 0.05). The RCS curves showed that there were non-linear relationships between rHGS levels and elevated blood pressure and hypertension (Figure 2). The relationship between rHGS levels and elevated blood pressure was “V” shaped, while the relationship between rHGS levels and hypertension was “U” shaped.

**Table 2:** Associations between HGS and elevated blood pressure and hypertension in children and adolescents.

Variables	Elevated blood pressure				Hypertension			
	Model 1		Model 2		Model 1		Model 2	
	OR (95%CI)	*P*	OR (95%CI)	*P*	OR (95%CI)	*P*	OR (95%CI)	*P*
rHGS								
Low	Ref		Ref		Ref		Ref	
Median	0.70 (0.47–1.05)	0.080	0.52 (0.32–0.85)	0.011	0.59 (0.37–0.95)	0.031	0.55 (0.34–0.89)	0.017
High	0.86 (0.67–1.11)	0.244	0.52 (0.33–0.81)	0.005	0.42 (0.25–0.73)	0.003	0.34 (0.18–0.66)	0.002
HGS asymmetry ratio								
0%–10%	Ref		Ref		Ref		Ref	
10.1%–20.0%	0.94 (0.69–1.28)	0.684	1.03 (0.75–1.42)	0.846	0.69 (0.44–1.07)	0.095	0.74 (0.47–1.17)	0.189
20.1%–30.0%	1.09 (0.64–1.86)	0.730	1.11 (0.66–1.87)	0.677	1.14 (0.53–2.45)	0.721	1.14 (0.57–2.27)	0.699
>30.0%	1.90 (1.13–3.20)	0.016	2.14 (1.27–3.61)	0.006	2.66 (1.11–6.37)	0.030	3.02 (1.42–6.42)	0.005

HGS, handgrip strength; rHGS, relative HGS [male (tertiles): <2.13 (low), 2.13–3.22 (median), ≥3.22 (high); female (tertiles): <1.86 (low), 1.86–2.4 (median), ≥2.4 (high)]; OR, odds ratio; CI, confidence interval; Ref, reference.

Model 1 is univariable logistic regression model.

Model 2 is multivariable logistic model adjusted for (1) elevated blood pressure: age, gender, race, education level, total cholesterol, HDL-C, cotinine, and BMI (not in analysis of rHGS); (2) hypertension: gender, race, education level, HDL-C, cotinine, and BMI (not in analysis of rHGS).

The relationships between HGS and elevated blood pressure and hypertension in children were further analyzed based on age (<13, ≥13 years) and gender (male, female) subgroups (Table 3). For rHGS, high rHGS levels were associated with lower odds of elevated blood pressure in children aged <13 years (OR = 0.34, 95%CI, 0.14–0.83) and ≥13 years (OR = 0.40, 95%CI, 0.23–0.69). High rHGS levels were also related to lower odds of hypertension in children aged <13 years (OR = 0.39, 95%CI, 0.16–0.94) and ≥13 years (OR = 0.26, 95%CI, 0.08–0.85). In the analysis of HGS asymmetry ratio, the association of HGS asymmetry ratio of >30.0% with higher odds of elevated blood pressure (OR = 2.54, 95%CI, 1.29–4.99) and hypertension (OR = 5.48, 95%CI, 1.78–16.87) was found only in children aged ≥13 years.

**Table 3:** Subgroup analysis of the relationships between HGS and elevated blood pressure and hypertension in age and gender subgroups.

Variables	Elevated blood pressure				Hypertension			
Age subgroups	Age <13 years		Age ≥13 years		Age <13 years		Age ≥13 years	
	OR (95%CI)	*P*	OR (95%CI)	*P*	OR (95%CI)	*P*	OR (95%CI)	*P*
rHGS								
Low	Ref		Ref		Ref		Ref	
Median	0.74 (0.48–1.14)	0.161	0.38 (0.22–0.68)	0.002	0.71 (0.46–1.10)	0.122	0.38 (0.13–1.06)	0.063
High	0.34 (0.14–0.83)	0.020	0.40 (0.23–0.69)	0.002	0.39 (0.16–0.94)	0.037	0.26 (0.08–0.85)	0.028
HGS asymmetry ratio								
0%–10%	Ref		Ref		Ref		Ref	
10.1%–20.0%	0.96 (0.65–1.41)	0.825	1.10 (0.71–1.71)	0.656	0.71 (0.37–1.37)	0.296	0.78 (0.39–1.57)	0.473
20.1%–30.0%	0.58 (0.26–1.30)	0.179	1.54 (0.87–2.71)	0.135	0.60 (0.23–1.60)	0.298	1.78 (0.72–4.39)	0.201
>30.0%	1.61 (0.48–5.42)	0.432	2.54 (1.29–4.99)	0.008	1.34 (0.33–5.40)	0.673	5.48 (1.78–16.87)	0.004
Gender subgroups	Males		Females		Males		Females	
	OR (95%CI)	*P*	OR (95%CI)	*P*	OR (95%CI)	*P*	OR (95%CI)	*P*
rHGS								
Low	Ref		Ref		Ref		Ref	
Median	0.58 (0.31–1.08)	0.086	0.36 (0.19–0.68)	0.002	0.66 (0.36–1.21)	0.170	0.33 (0.18–0.62)	<.001
High	0.56 (0.32–0.99)	0.045	0.34 (0.20–0.55)	<.001	0.34 (0.16–0.71)	0.006	0.28 (0.10–0.81)	0.021
HGS asymmetry ratio								
0%–10%	Ref		Ref		Ref		Ref	
10.1%–20.0%	1.14 (0.80–1.63)	0.461	0.90 (0.53–1.51)	0.675	0.74 (0.42–1.31)	0.294	0.73 (0.39–1.35)	0.303
20.1%–30.0%	1.46 (0.85–2.50)	0.168	0.73 (0.27–1.95)	0.518	1.74 (0.93–3.27)	0.083	0.40 (0.09–1.90)	0.241
>30.0%	2.91 (1.36–6.20)	0.007	1.36 (0.46–4.04)	0.566	4.74 (1.69–13.24)	0.004	1.01 (0.25–4.10)	0.986

HGS, handgrip strength; rHGS, relative HGS [male (tertiles): <2.13 (low), 2.13–3.22 (median), ≥3.22 (high); female (tertiles): <1.86 (low), 1.86–2.4 (median), ≥2.4 (high)]; OR, odds ratio; CI, confidence interval; Ref, reference.

All analyses were multivariable logistic models adjusted for (1) elevated blood pressure: age (not in age subgroup), gender (not in gender subgroup), race, education level, total cholesterol, HDL-C, cotinine, and BMI (not in analysis of rHGS); (2) hypertension: gender (not in gender subgroup), race, education level, HDL-C, cotinine, and BMI (not in analysis of rHGS).

In the gender subgroups, high rHGS levels were related to lower odds of elevated blood pressure and hypertension in males [elevated blood pressure: OR = 0.56, 95%CI, 0.32–0.99; hypertension: OR = 0.34, 95%CI, 0.16–0.71] and females [elevated blood pressure: OR = 0.34, 95%CI, 0.20–0.55; hypertension: OR = 0.28, 95%CI, 0.10–0.81]. Moreover, the relationship of HGS asymmetry ratio of >30.0% with higher odds of elevated blood pressure (OR = 2.91, 95%CI, 1.36–6.20) and hypertension (OR = 4.74, 95%CI, 1.69–13.24) was observed only in males.

## Discussion

The current study analyzed the relationship between HGS and elevated blood pressure and hypertension in children and adolescents. Our results showed that high rHGS levels were correlated with lower odds of elevated blood pressure and hypertension in children. HGS asymmetry ratio of >30.0% were related to higher odds of elevated blood pressure and hypertension in children. Subgroup analysis found that the relationship between high rHGS levels and lower odds of elevated blood pressure and hypertension was observed in children aged <13 years or ≥13 years and males or females, whereas the association between HGS asymmetry ratio of >30.0% and higher odds of elevated blood pressure and hypertension was found only in children ≥13 years and males.

Muscle strength is a good indicator of overall health, and HGS is a reliable biomarker for assessing muscle strength (19, 20). Previous studies have reported a relationship between HGS and blood pressure in adults (8, 9, 21). Increased HGS was significantly associated with a reduced risk of hypertension in adult women (8). A prospective cohort study showed that high weight-adjusted HGS was associated with a lower risk of hypertension in middle-aged and older adults, but not absolute HGS (9). Several meta-analyses have shown isometric HGS training to be effective in reducing resting blood pressure in adults (22, 23). Some studies have also reported a relationship between HGS and other diseases such as pulmonary hypertension (24), cardiovascular disease (25), and chronic kidney disease (26). HGS decreased with the increase of the level of forebrain natriuretic peptide, a key indicator of pulmonary hypertension monitoring (24). In addition, BMI may affect the role of HGS (12, 27), and the association between blood pressure and handgrip strength in children may be confused by BMI (10). This study assessed the relationship between BMI-adjusted HGS (rHGS) and elevated blood pressure and hypertension in children aged 6–19 years. High rHGS levels were found to be correlated with lower odds of elevated blood pressure and hypertension in children. Our findings suggested that the relationship between HGS and blood pressure in children was consistent with the results of previous studies in adults (8, 9). In addition, an HGS asymmetry ratio of >30.0% was associated with higher odds of elevated blood pressure and hypertension. The HGS asymmetry ratio reflects the difference of HGS between the two hands of an individual, and a large HGS asymmetry ratio may represent a functional deficiency of the neuromuscular system (14). Moreover, subgroup analysis showed that the relationship between HGS asymmetry ratio of >30.0% and higher odds of elevated blood pressure and hypertension was observed only in children ≥13 years and males. A study also showed no difference in HGS between males and females from 6 to 11 years of age, but from 12 years of age onwards, males had higher HGS values in both hands (28).

The exact mechanism by which HGS affects blood pressure in children remains unclear, and several possible explanations have been proposed in previous studies. Possible mechanisms for the effects of HGS training on blood pressure include improvements in conduit and resistance due to endothelium-dependent dilation, oxidative stress, and autoregulation of heart rate and blood pressure (29). HGS training may lower blood pressure by attenuating peripheral sympathetic vasoconstrictor activity (30). HGS training is associated with increased NO-dependent dilation of local ductus arteriosus in hypertensive patients (31). Moreover, HGS training improves the vasodilatory response, and prolonged training may increase vessel diameter and decrease total peripheral resistance, which in turn enhances autonomic vasodilation (32, 33). In addition to representing muscle strength, HGS is also an indicator of muscle mass and even nutritional status (19). Individuals with higher HGS levels are more likely to engage in healthy lifestyle behaviors that affect hypertension risk and overall health. Improved muscle health is associated with the release of cytokines and myokines into the circulation, which may enhance anti-atherosclerotic properties (34, 35). However, the specific mechanisms underlying the effects of HGS on blood pressure in children may depend on subsequent mechanistic studies.

We assessed the relationship between HGS and elevated blood pressure and hypertension in children and adolescents. Unlike previous studies in adults, we used rHGS and HGS asymmetry ratio instead of absolute HGS. rHGS can rule out the effect of BMI on HGS. Nevertheless, several limitations of this study should be considered. First, this study was a cross-sectional study design that did not allow for causal inferences and may be subject to some bias. The association between HGS-related indicators and hypertension in children and adolescents still needs further validation in prospective studies. Second, judgments of elevated blood pressure and hypertension in this study were based on multiple blood pressure measurements taken on a single day, rather than spread over two or more visits, which may have been biased. Third, some potential confounders such as family history of hypertension could not be obtained due to limitations of the NHANES database.

## Conclusions

High rHGS levels were related to lower odds of elevated blood pressure and hypertension in children and adolescents, whereas an HGS asymmetry ratio of >30.0% was correlated with higher odds of elevated blood pressure and hypertension. In addition, the association between rHGS and elevated blood pressure and hypertension did not differ by age or sex, whereas the correlation between HGS asymmetry ratio and elevated blood pressure and hypertension was found only in children ≥13 years and males.

## Data Availability

Publicly available datasets were analyzed in this study. This data can be found here: NHANES database, https://wwwn.cdc.gov/nchs/nhanes/.
