# Supplementary material for: Association between handgrip strength and hypertension in children and adolescents: an analysis of the National Health and Nutrition Examination Survey 2011–2014
Source: Front Pediatr. 2025 Apr 14;13:1559556. doi: 10.3389/fped.2025.1559556 (PMC12034725; doi:10.3389/fped.2025.1559556)
Supplement: Supplementary file 1 [file Table1.docx]

**Supplement Table 1**. Difference analysis before and after interpolation of missing values.

| **Variables** | **Missing (%)** | **After interpolation** | **Before interpolation** | **Statistics** | ***P*** |
| --- | --- | --- | --- | --- | --- |
| Education level, n (%) | 3 (0.08) |  |  | χ^2^=1.339 | 0.512 |
| Below high school |  | 3378 (89.05) | 3376 (89.07) |  |  |
| High school |  | 198 (5.85) | 198 (5.85) |  |  |
| Above high school |  | 160 (5.10) | 159 (5.08) |  |  |
| Physical activity, n (%) | 38 (1.02) |  |  | χ^2^=0.176 | 0.675 |
| Not Ideal physical activity |  | 1392 (35.11) | 1379 (35.16) |  |  |
| Ideal physical activity |  | 2344 (64.89) | 2319 (64.84) |  |  |
| Direct HDL-C (mg/dL), Mean (S.E) | 491 (13.14) | 52.09 (0.37) | 51.98 (0.40) | t=0.97 | 0.340 |
| Total cholesterol (mg/dL), Mean (S.E) | 491 (13.14) | 157.05 (0.70) | 157.14 (0.78) | t=-0.31 | 0.757 |
| HEI-2020, Mean (S.E) | 274 (7.33) | 46.34 (0.37) | 46.26 (0.36) | t=0.91 | 0.371 |

Note: HDL-C, high density lipoprotein cholesterol; HEI-2020, Healthy Eating Index-2020.

**Supplement Table 2**. Confounders associated with elevated blood pressure screened by weighted univariable logistic regression model.

| **Variables** | **OR (95%CI)** | **P** |
| --- | --- | --- |
| Age | 1.08 (1.04-1.12) | <0.001 |
| Gender |  |  |
| Male | Ref |  |
| Female | 0.43 (0.32-0.57) | <0.001 |
| Race |  |  |
| White | Ref |  |
| Black | 1.49 (1.15-1.94) | 0.004 |
| Others | 1.06 (0.77-1.46) | 0.719 |
| Education level |  |  |
| Below high school | Ref |  |
| High school | 1.71 (1.08-2.72) | 0.023 |
| Above high school | 1.90 (0.90-4.01) | 0.091 |
| PIR |  |  |
| <1.3 | Ref |  |
| ≥1.3 | 0.81 (0.63-1.03) | 0.086 |
| Unknown | 0.75 (0.42-1.34) | 0.322 |
| Screen time |  |  |
| <5 | Ref |  |
| ≥5 | 1.22 (0.86-1.73) | 0.260 |
| Physical activity |  |  |
| Not Ideal physical activity | Ref |  |
| Ideal physical activity | 1.08 (0.86-1.36) | 0.493 |
| Birth weight |  |  |
| <5.5 | Ref |  |
| ≥5.5 | 0.63 (0.36-1.12) | 0.114 |
| Unknown | 1.41 (0.82-2.44) | 0.208 |
| Household Smokers |  |  |
| No | Ref |  |
| Yes | 1.42 (0.98-2.06) | 0.064 |
| Unknown | 1.20 (0.72-2.00) | 0.468 |
| Family education |  |  |
| Below high school | Ref |  |
| High school | 1.10 (0.76-1.58) | 0.605 |
| Above high school | 0.86 (0.62-1.19) | 0.353 |
| Unknown | 1.30 (0.58-2.95) | 0.511 |
| Total cholesterol | 1.01 (1.01-1.01) | 0.040 |
| Direct HDL-C | 0.98 (0.97-0.99) | <0.001 |
| Cotinine |  |  |
| <0.05 | Ref |  |
| ≥0.05 | 1.79 (1.39-2.32) | <0.001 |
| Unknown | 0.94 (0.54-1.63) | 0.809 |
| Body Mass Index | 1.08 (1.06-1.09) | <0.001 |
| HEI-2020 | 1.00 (0.98-1.01) | 0.627 |

Note: PIR, family poverty-to-income ratio; HDL-C, high density lipoprotein cholesterol; HEI-2020, Healthy Eating Index-2020; Ref, reference; CI, confidence interval.

**Supplement Table 3**. Confounders associated with hypertension screened by weighted univariable logistic regression model.

| **Variables** | **OR (95%CI)** | **P** |
| --- | --- | --- |
| Age | 1.00 (0.94-1.06) | 0.996 |
| Gender |  |  |
| Male | Ref |  |
| Female | 0.56 (0.33-0.94) | 0.030 |
| Race |  |  |
| White | Ref |  |
| Black | 1.67 (1.13-2.46) | 0.012 |
| Others | 1.30 (0.74-2.26) | 0.347 |
| Education level |  |  |
| Below high school | Ref |  |
| High school | 2.59 (1.38-4.87) | 0.004 |
| Above high school | 0.78 (0.42-1.44) | 0.418 |
| PIR |  |  |
| <1.3 | Ref |  |
| ≥1.3 | 0.68 (0.43-1.06) | 0.084 |
| Unknown | 1.15 (0.59-2.24) | 0.667 |
| Screen time |  |  |
| <5 | Ref |  |
| ≥5 | 1.33 (0.89-1.99) | 0.156 |
| Physical activity |  |  |
| Not Ideal physical activity | Ref |  |
| Ideal physical activity | 0.94 (0.65-1.37) | 0.747 |
| Birth weight |  |  |
| <5.5 | Ref |  |
| ≥5.5 | 0.76 (0.42-1.38) | 0.360 |
| Unknown | 1.23 (0.67-2.25) | 0.489 |
| Household Smokers |  |  |
| No | Ref |  |
| Yes | 1.30 (0.84-2.02) | 0.235 |
| Unknown | 1.01 (0.66-1.53) | 0.978 |
| Family education |  |  |
| Below high school | Ref |  |
| High school | 1.05 (0.54-2.05) | 0.878 |
| Above high school | 0.68 (0.40-1.14) | 0.139 |
| Unknown | 1.11 (0.36-3.39) | 0.851 |
| Total cholesterol | 1.00 (0.99-1.01) | 0.579 |
| Direct HDL-C | 0.98 (0.96-0.99) | 0.001 |
| Cotinine |  |  |
| <0.05 | Ref |  |
| ≥0.05 | 2.33 (1.35-4.01) | 0.003 |
| Unknown | 1.18 (0.55-2.51) | 0.667 |
| Body Mass Index | 1.08 (1.06-1.10) | <0.001 |
| HEI-2020 | 0.99 (0.97-1.01) | 0.490 |

Note: PIR, family poverty-to-income ratio; HDL-C, high density lipoprotein cholesterol; HEI-2020, Healthy Eating Index-2020; Ref, reference; CI, confidence interval.

**Supplement Table 4**. Characteristics of children and adolescents according to hypertension.

| **Variables** | **Total (n=3736)** | **Non-hypertension (n=3548)** | **Hypertension (n=188)** | ***P*** |
| --- | --- | --- | --- | --- |
| Age (years), Mean (S.E) | 13.44 (0.10) | 13.44 (0.10) | 13.44 (0.38) | 0.996 |
| Gender, n (%) |  |  |  | 0.022 |
| Male | 1876 (50.28) | 1759 (49.60) | 117 (63.91) |  |
| Female | 1860 (49.72) | 1789 (50.40) | 71 (36.09) |  |
| Race, n (%) |  |  |  | 0.116 |
| White | 932 (54.92) | 892 (55.33) | 40 (46.73) |  |
| Black | 1015 (14.22) | 953 (13.95) | 62 (19.63) |  |
| Others | 1789 (30.86) | 1703 (30.72) | 86 (33.64) |  |
| Education level, n (%) |  |  |  | <.001 |
| Below high school | 3378 (89.05) | 3222 (89.35) | 156 (83.04) |  |
| High school | 199 (5.87) | 177 (5.51) | 22 (13.21) |  |
| Above high school | 159 (5.08) | 149 (5.14) | 10 (3.75) |  |
| PIR, n (%) |  |  |  | 0.095 |
| <1.3 | 1606 (31.94) | 1516 (31.59) | 90 (39.10) |  |
| ≥1.3 | 1861 (61.93) | 1781 (62.41) | 80 (52.34) |  |
| Unknown | 269 (6.12) | 251 (6.00) | 18 (8.56) |  |
| Screen time (hours) |  |  |  | 0.145 |
| <5 | 2573 (70.97) | 2452 (71.26) | 121 (65.06) |  |
| ≥5 | 1163 (29.03) | 1096 (28.74) | 67 (34.94) |  |
| Physical activity, n (%) |  |  |  | 0.684 |
| Non-ideal | 1397 (35.27) | 1325 (35.19) | 72 (36.87) |  |
| Ideal | 2339 (64.73) | 2223 (64.81) | 116 (63.13) |  |
| Birth weight (pounds), n (%) |  |  |  | 0.005 |
| <5.5 | 310 (6.28) | 294 (6.26) | 16 (6.68) |  |
| ≥5.5 | 2247 (59.46) | 2153 (59.98) | 94 (48.95) |  |
| Unknown | 1179 (34.27) | 1101 (33.76) | 78 (44.37) |  |
| Household Smokers, n (%) |  |  |  | 0.353 |
| No | 3058 (83.00) | 2909 (83.13) | 149 (80.46) |  |
| Yes | 459 (11.09) | 431 (10.96) | 28 (13.78) |  |
| Unknown | 219 (5.91) | 208 (5.91) | 11 (5.76) |  |
| Family education, n (%) |  |  |  | 0.280 |
| Below high school | 925 (19.28) | 873 (19.10) | 52 (23.07) |  |
| High school | 828 (20.85) | 784 (20.58) | 44 (26.16) |  |
| Above high school | 1866 (56.85) | 1781 (57.35) | 85 (46.79) |  |
| Unknown | 117 (3.02) | 110 (2.97) | 7 (3.99) |  |
| Total cholesterol (mg/dL), Mean (S.E) | 157.39 (0.69) | 157.39 (0.71) | 157.42 (2.76) | 0.992 |
| Direct HDL-C (mg/dL), Mean (S.E) | 52.06 (0.36) | 52.19 (0.38) | 49.42 (0.90) | 0.006 |
| Cotinine (μg/L), n (%) |  |  |  | 0.002 |
| <0.05 | 1869 (52.10) | 1794 (52.91) | 75 (35.61) |  |
| ≥0.05 | 1382 (35.87) | 1287 (34.94) | 95 (54.79) |  |
| Unknown | 485 (12.03) | 467 (12.15) | 18 (9.61) |  |
| HEI-2020, Mean (S.E) | 46.18 (0.33) | 46.25 (0.33) | 44.66 (1.31) | 0.227 |
| HGS, Mean (S.E) | 28.38 (0.37) | 28.25 (0.35) | 30.95 (1.39) | 0.037 |
| HGS, n (%) |  |  |  | 0.088 |
| Low | 1405 (32.90) | 1339 (32.83) | 66 (34.36) |  |
| Median | 1240 (33.76) | 1191 (34.15) | 49 (25.86) |  |
| High | 1091 (33.34) | 1018 (33.02) | 73 (39.78) |  |
| rHGS, Mean (S.E) | 2.44 (0.03) | 2.45 (0.03) | 2.29 (0.07) | 0.019 |
| rHGS, n (%) |  |  |  | 0.002 |
| Low | 1409 (33.00) | 1317 (32.24) | 92 (48.37) |  |
| Median | 1245 (33.97) | 1195 (34.16) | 50 (30.22) |  |
| High | 1082 (33.03) | 1036 (33.61) | 46 (21.41) |  |
| HGS asymmetry ratio, n (%) |  |  |  | 0.031 |
| 0-10% | 2206 (59.62) | 2094 (59.49) | 112 (62.23) |  |
| 10.1%-20.0% | 1112 (29.16) | 1066 (29.56) | 46 (21.21) |  |
| 20.1%-30.0% | 320 (8.86) | 300 (8.78) | 20 (10.50) |  |
| >30.0% | 98 (2.36) | 88 (2.18) | 10 (6.06) |  |

Note: PIR, family poverty-to-income ratio; HDL-C, high density lipoprotein cholesterol; HEI-2020, Healthy Eating Index-2020; HGS, handgrip strength; rHGS, relative HGS [male (tertiles): <2.13 (low), 2.13-3.22 (median), ≥3.22 (high); female (tertiles): <1.86 (low), 1.86-2.4 (median), ≥2.4 (high)].
